# Supplementary material for: Reconciling Mining with the Conservation of Cave Biodiversity: A Quantitative Baseline to Help Establish Conservation Priorities
Source: PLoS One. 2016 Dec 20;11(12):e0168348. doi: 10.1371/journal.pone.0168348 (PMC5173368; doi:10.1371/journal.pone.0168348)
Supplement: S1 Dataset — (ZIP) [file pone.0168348.s002.zip › Taxa/Serra Norte/SN_2013/N4WS/Lista N4WS-08.pdf]

CAVIDADE N4WS-0008

Classe	Ordem	Fam/Outros	Gên/Outros	Espécie	S-U	U-S	Zon.
Annelida	Oligochaeta			sp.2	32	2	P
Arachnida	Acari	(Oribatida)		sp.1	X	X	P
Arachnida	Acari	(Oribatida)		sp.2	X		P
Arachnida	Acari	(Parasitengonina)		sp.1	X	X	P/A
Arachnida	Acari	(Parasitengonina)		sp.2		X	P
Arachnida	Acari	(Uropodina)		sp.1	X		A
Arachnida	Acari	Acaridae		jovem	X		A
Arachnida	Acari	Anystidae	*Erythracarus*	*nasutus*		X	P
Arachnida	Acari	Cunaxidae		sp.1	X	X	P/A
Arachnida	Acari	Macronyssidae		sp.1	X	X	P/A
Arachnida	Acari	Mesostigmata		sp.1	X	X	P
Arachnida	Acari	Podocinidae	*Podocinum*	sp.1	X		P
Arachnida	Acari	Prostigmata		sp.1	X		P
Arachnida	Acari	Pyrosejidae		sp.1		X	P
Arachnida	Acari	Stigmaeidae		sp.1		X	P
Arachnida	Acari	Tarsonemidae		sp.1	X		A
Arachnida	Amblypygi	Charinidae	*Charinus*	sp.1	25	2	P/A
Arachnida	Amblypygi	Phrynidae	*Heterophrynus*	*longicornis*	19	13	E/P/A
Arachnida	Araneae	Araneidae		jovem	X		E
Arachnida	Araneae	Ctenidae	*Ctenus*	sp.3	5	7	E/P
Arachnida	Araneae	Ochyroceratidae	*Ochyrocera*	sp.1	X		A
Arachnida	Araneae	Ochyroceratidae	*Speocera*	sp.1	X	X	P/A
Arachnida	Araneae	Scytodidae	*Scytodes*	*globula*	7	8	E/P
Arachnida	Araneae	Theraphosidae	aff. *Holothele*	sp.1		3	E/P
Arachnida	Araneae	Theridiidae	*Achaearanea*	sp.1	X	X	P
Arachnida	Araneae	Theridiosomatidae	*Plato*	sp.1	X	X	E/P
Arachnida	Opiliones	Escadabiidae		sp.2	X	X	P/A
Arachnida	Pseudoscorpiones	Chernetidae	*Spelaeochernes*	sp.1	X	X	P/A
Arachnida	Pseudoscorpiones	Chthoniidae	*Pseudochthonius*	sp.1	X	X	P/A
Arachnida	Schizomida	Hubbardiidae	aff. *Rowlandius*	sp.1	X	X	P/A
Chilopoda	Geophilomorpha	Geophilidae	*Hyphydrophilus*	sp.1	3	7	P
Chilopoda	Scolopendromorpha	Scolopocryptopidae	*Dinocryptops*	*miersii*		2	A
Chilopoda	Scutigeromorpha	Pselliodidae	*Sphendononema*	*guildingii*	1		E
Diplopoda	Polydesmida	Pyrgodesmidae		sp.1		X	P/A
Diplopoda	Polyxenida	Hypogexenidae		sp.1		X	P
Diplopoda	Spirostreptida	Pseudonannolenidae	*Pseudonannolene*	sp.1		3	P
Entognatha	Collembola	Cyphoderidae		sp.1		X	P
Entognatha	Collembola	Isotomidae		sp.1	X	X	P/A
Entognatha	Collembola	Paronellidae		sp.1		X	P
Entognatha	Collembola	Symphypleona		sp.1	X	X	P
Entognatha	Diplura	Japygidae		sp.1		X	P
Insecta	Blattodea	Blattellidae		sp.3	5		A
Insecta	Blattodea	Blattidae		jovem		1	P
Insecta	Coleoptera	Carabidae		sp.21	X	X	P
Insecta	Coleoptera	Curculionidae	Scolytinae	sp.2		X	E
Insecta	Coleoptera	Dytiscidae		sp.11B		X	P
Insecta	Coleoptera	Dytiscidae		sp.3	X	X	P
Insecta	Coleoptera	Elateridae		jovem		X	P
Insecta	Coleoptera	Hydrophilidae	Sphaeridiinae	sp.13		X	A

Insecta	Coleoptera	Staphylinidae		sp.35	X		P
Insecta	Coleoptera	Staphylinidae		sp.40	X		P
Insecta	Coleoptera	Staphylinidae		sp.68		X	P
Insecta	Coleoptera	Tenebrionidae		jovem	X		P
Insecta	Diptera	Cecidomyiidae	Cecidomyiinae	sp.		X	P
Insecta	Diptera	Ceratopogonidae		jovem	X		P
Insecta	Diptera	Culicidae		jovem		X	P/A
Insecta	Diptera	Drosophilidae	*Drosophila*	*eleonorae*		X	P
Insecta	Diptera	Milichiidae	*Milichia*	sp.		X	A
Insecta	Diptera	Muscidae		jovem		X	P
Insecta	Diptera	Phoridae	Metopininae	sp.		X	P
Insecta	Diptera	Psychodidae	*Pintomyia*	*gruta*	X		P
Insecta	Diptera	Psychodidae	*Sciopemyia*	*sordellii*	X	X	P/A
Insecta	Diptera	Psychodidae	*Telmatoscopus*	sp.	X		P
Insecta	Diptera	Tipulidae	Tipulinae	sp.	X		E
Insecta	Heteroptera	Cydnidae	*Pangaeus*	sp.1	X	X	P/A
Insecta	Heteroptera	Reduviidae	Emesinae	sp.4	1		P
Insecta	Heteroptera	Reduviidae	Reduviinae	jovem	2	2	P
Insecta	Heteroptera	Schizopteridae	Schizopterinae	sp.1	X	X	P/A
Insecta	Heteroptera	Veliidae	*Paravelia*	sp.1	X	X	P
Insecta	Hymenoptera	Diapriidae		sp.1	X		P
Insecta	Hymenoptera	Diapriidae		sp.2	X	X	P/A
Insecta	Hymenoptera	Formicidae	*Gnamptogenys*	*striatula*		X	P
Insecta	Hymenoptera	Formicidae	*Pachycondyla*	*striata*	3	3	P
Insecta	Hymenoptera	Formicidae	*Solenopsis*	sp.1	X		P/A
Insecta	Hymenoptera	Formicidae	*Solenopsis*	sp.2		X	P/A
Insecta	Hymenoptera	Formicidae	*Wasmannia*	sp.1	X		P
Insecta	Lepidoptera	Tineoidea		sp.1	X	X	P/A
Insecta	Lepidoptera	Tineoidea		sp.2	X		P
Insecta	Orthoptera	Phalangopsidae	*Paraclodes*	sp.	2		E
Insecta	Orthoptera	Phalangopsidae	*Phalangopsis*	sp.	735	100	P/A
Insecta	Thysanura	Nicoletiidae		sp.1	X	X	P/A
Gastropoda	Pulmonata	Subulinidae	*Lamellaxis*	sp.		X	P
Gastropoda	Pulmonata	Subulinidae	*Lamellaxis*	sp.		X	P
Malacostraca	Isopoda	Philosciidae		sp.1	X	X	A
Malacostraca	Isopoda	Philosciidae		sp.2		X	P
Nematomorpha				sp.1	1	1	A
Amphibia	Anura	Leptodactylidae	*Leptodactylus*	cf. *vastus*	2	1	P
Amphibia	Anura	Strabomantidae	*Pristimantis*	cf. *fenestratus*		4	P
Mammalia	Chiroptera	Emballonuridae	*Peropteryx*	sp.		5	P
Mammalia	Chiroptera	Furipteridae	*Furipterus*	*horrens*	6		P
Mammalia	Chiroptera	Phyllostomidae	*Carollia*	*perspicillata*	50	70	P
Mammalia	Chiroptera	Phyllostomidae	*Diphylla*	*ecaudata*	2	3	P
Mammalia	Chiroptera	Phyllostomidae	*Lonchorrhina*	sp.	12	70	P
Mammalia	Chiroptera	Phyllostomidae	*Trachops*	*cirrhosus*	1	1	P
Mammalia	Rodentia	Cricetidae	*Rhipidomys*	sp.	2		P
